# Analysis of Tribological Properties of Hardfaced High-Chromium Layers Subjected to Wear in Abrasive Soil Mass

**DOI:** 10.3390/ma17143461

**Published:** 2024-07-12

**Authors:** Magdalena Lemecha, Krzysztof Ligier, Jerzy Napiórkowski, Oleksandr Vrublevskyi

**Affiliations:** Department of Construction and Operation of Vehicles and Machines, Faculty of Technical Sciences, University of Warmia and Mazury in Olsztyn, 10-719 Olsztyn, Poland; krzysztof.ligier@uwm.edu.pl (K.L.); jerzy.napiorkowski@uwm.edu.pl (J.N.); aleksander.wroblewski@uwm.edu.pl (O.V.)

**Keywords:** AR steel, padding weld Fe–C–Cr, abrasive wear, rotating bowl method, computer image analysis

## Abstract

This article presents the results of abrasion wear resistance tests of wear-resistant steel and surfacing under laboratory conditions and natural operation. Abrasion wear resistance determined on the basis of the study by determining geometrical characteristics of the alloying additives using computer image analysis methods, as well as examining the changes occurring on the surface of the workpieces and their wear intensity. Based on the results obtained from laboratory tests, it was noted that AR steel exhibited 14 times greater wear than the padding weld. This wear is affected by alloy additives, which, for the padding weld, are chromium additives. The microstructure image shows that soil mass had a destructive effect mainly on the matrix of the material, whereas in the areas with high concentrations of chromium precipitates, this effect was significantly weaker. The operational test results showed that within the area of the tine subjected to hardfacing, the material loss was lower than that for the same area of the tine in the as-delivered state. For the hardfaced tine, a 7% loss of volume was noted in relation to the operating part before testing and following the friction process. However, for the operating part in the as-delivered state, this difference amounted to 12%.

## 1. Introduction

Technologies for extracting and transporting minerals or processing soil are the same in most industrialised countries. Essentially, the concept of soil-processing machinery has been unchanged for many years. What has been changing are the technological and design forms of operating parts interacting directly with the soil mass. For this reason, despite the many studies already available, the assessment of their durability by learning about and describing the phenomena generated by the interaction between the operating part and the soil is still of fundamental importance. This is due to the fact that, to date, universal and common technologies have not been developed to ensure the durability of working elements at an adequate level.

In recent years, many methods have been developed to analyse data on local loads on soil-processing operating parts in terms of the selection of materials [1]. Research has been carried out on the pressure distribution between the soil and the operating part, particularly using analytical models based on soil pressure theory [2]. Numerical models have also been developed to analyse the wear process and optimise the impact of cutting tools on the soil [3,4,5,6].

Bentaher et al. [3] used the finite element method (FEA) to model the soil cutting process. The influence of the cutting angle (the angle between the horizontal matrix and the cutting direction) and the lift angle (the angle between the surface of the board and the horizontal line in the section perpendicular to the cutting edge) on the thrust force was investigated. Their numerical results showed good agreement with experimental results reported in the literature. The developed numerical model was judged to be useful for designing tools adapted to different type of soil conditions.

The authors of another paper [4] compared the performance of different drill coulter designs in different soils using the DEM method.

The modelling of tribological interactions between working bodies and the substrate using 3D scanning technology is presented in [5]. The suitability of the method used was confirmed by comparing it with the mass wear assessment method and the results of applying the proposed method for different abrasive wear conditions of working parts.

Another study [6] presents numerical modelling of the tillage process. The modelling uses the DEM method to model and calibrate the physical and mechanical properties of the abrasive medium. Based on the simulation of the process, the components of the traction resistance of the plough body were obtained.

The authors indicate that the obtained dependence of the plough body traction resistance components on soil moisture and surface energy facilitates the selection of parameters of the Hertz–Mindlin contact model when modelling the behaviour of the soil environment during interaction with the working tools of tillage machines.

The use of 3D scanning to assess the wear course has proven helpful in this regard [7]. Despite efforts to optimise the shape of soil-processing operating parts, it has still not been possible to eliminate their intensive wear [8]. The wear intensity is mainly affected by the properties of the abrasive soil mass, including the soil grain size distribution, soil moisture content, the shape of abrasive particles, hardness of the tool material, and the distribution of pressure between the soil and the tool [9,10,11]

The author of the paper [9] indicates that the finite element method can be used to predict changes in the geometry of a tool’s cutting edge and to predict soil deformation.

In [10], the authors present a study of the intensity of abrasive wear of surfaced layers under varying soil conditions. The authors found that the course of wear of surfaced layers in soil masses with a high content of loose abrasive grains is determined by the size of carbide precipitates.

The authors [11] indicate that the use of surfaced layers 3 times reduced plough wear and 1.5 times reduced plough chisels. The experimental results obtained were implemented in the Holm–Archard model. According to the authors [11], the proposed model can be used to predict the durability of tillage tools.

The choice of materials for operating parts interacting with the soil has been one of the main factors limiting the functional capabilities of machines since the beginning of the machinery design process. The durability of individual operating parts under varying soil conditions shows considerable dispersion and is determined not only by design solutions but also by the types of materials used and their heat treatment. The aim of reducing the material consumption index of operating parts exposed to abrasive wear while maintaining their durability is achievable by reducing the intensity of destructive phenomena occurring in materials. This involves learning about and describing the relationships between the chemical composition, microstructure, and properties of materials; developing research methods that model wear phenomena well; and developing methods for describing phenomena and predicting durability.

The expectations placed on operating parts exposed to abrasive wear can be expressed as follows:

(a) High resistance to wear, including abrasive wear;
(b) Capacity to transfer variable loads;
(c) Development of grain geometry;
(d) Joinability by welding techniques.

The following groups of steels can be distinguished among the modern structural materials used for operating parts of machines and tools [12,13,14,15,16]:

- Steels resistant to abrasive wear (known as abrasion-resistant steels) [12,13];
- Boron steels [14,15];
- Vanadium steels [16].

One study [12] tested the wear resistance of five types of steel (S235Jr, S355J2, C45, AISI 304, and Hardox 500) using the dry sand–rubber wheel method. When silicon carbide was used as an abrasive, Hardox 500 steel showed a 0 20% higher wear resistance index value than C45 steel. Under operating conditions, Hardox 500 steel showed lower weight loss than perlitic steel extracted from recycled railroad rails [13].

Another paper [14] compared two types of steels referred to as wear-resistant steels on abrasive wear. The wear of a newly developed high-strength low-alloy steel (HSLA) with manganese, chromium, nickel, and molybdenum content was compared with that of Hardox 400 steel. The authors found that the newly developed HSLA steel showed one-third the wear rate of Hardox 400 steel.

Hardox steels contain boron in their chemical composition, which affects their anti-wear properties. The effect of boron in the presence of chromium, vanadium, and titanium on wear resistance was studied in [15]. The paper showed that the improvement of wear resistance can be aided by the combined addition of chromium, boron, and vanadium. In the case of unalloyed or chromium steels, the boron micro-additive does not provide benefits in improving tribological properties.

One paper [16] presents the results of abrasive wear tests of Vanadis 60 SuperClean powder steel in comparison with Hardox 600 steel and PMFe60P surfacing. The study was conducted employing the “spinning bowl” method using natural abrasive ground masses. The results show that the weight loss of Vanadis 60 SuperClean powder steel in both types of abrasive ground was about seven times lower than that of Hardox 600 steel and two times lower than that of PMFe60P surfacing. The powder steel’s high resistance to abrasive wear in the abrasive ground masses was related to the presence of a large number of fine tungsten carbide and vanadium precipitates in its microstructure.

Additionally, for surfaces exposed to the greatest wear, refs. [17,18,19,20,21] the following are used:

- Multilayer weld deposits based on transition metals and amphoteric elements;
- Sintered carbides.

The concept of using wear-resistant steels [20] and multilayer cutting blades does not always meet users’ expectations.

In [17], it was shown that steel abrasive wear values change with changes in soil density. For a large friction surface, the water contained in it becomes a lubricant, thus contributing to the stabilisation of the friction process and to a reduction in wear. Therefore, working parts for soil processing are commonly used as replaceable elements [18]. This problem results from the inappropriateness of technological designs and forms for the environmental data of soil processing. Production plants produce consumable parts adapted to universal processing conditions. Only the direct user can, within the limits of their technical capabilities, adapt the technological form of the cutout to environmental conditions. Such activities will be cost effective and technologically feasible if the user has access to appropriate supporting materials [19]. The basic technology for increasing the wear resistance of such working parts is hardfacing using a coated electrode. This electrode consists of a core with a shell surrounding it. The overlay can be made by arc welding. This type of layering technology is widely available. The properties of the layer applied in this way are determined by the chemical composition of the electrode and the application parameters [20]. Another study [21] presents research on the wear intensity of welded layers in various ground conditions. The research was carried out using three types of soil masses. The wear analysis shows that of all the tested abrasive masses, the most wear-resistant were welding layers containing niobium and chromium carbides as well as molybdenum and vanadium carbides. Fe–C–Cr layers containing only niobium do not provide high wear resistance in the soil mass. Among the available additive materials, those with chromium additives are most often used for hardfacing and regeneration [22].

The ploughshares and cultivator tines are among the soil-processing parts that experience the highest wear (per unit and total). Fe–C–Cr alloys have sub-eutectic, eutectic, and super-eutectic structures [23], which provide good wear resistance [24]. The primary M_7_C_3_ carbides are formed at high carbon concentrations. The Fe–C–Cr alloys, with high chromium content, are used to make operating parts resistant to severe abrasive conditions. The microstructure of these alloys contains M_23_C_6_ carbides, which are composites with large and hard carbides in a softer Cr–Fe alloy core matrix.

The current study analysed the effect of soil conditions on the development of wear on the steel used for soil-processing operating parts and the surface layer applied to it using hardfacing with an Fe–C–Cr-based binder.

The novelty in this work is the determination of wear characteristics in the volumes bounded by the incisal planes of the tine. For the first time, it is proposed to make the position of the incisal planes dependent on the soil cultivation conditions (angle of the tine in the cultivator under real conditions). This made it possible to take into account the technological factors influencing wear. For this purpose, mesh surface (shell) models based on 3D scanning results were used. The results obtained from in-service testing and modelling were compared with the results of wear tests under laboratory conditions. An additional aspect is the analysis of the shape of the chromium carbides in the microstructure and the evaluation of its influence on the obtained wear values.

## 2. Materials and Methods

### 2.1. Materials

For the laboratory testing, an abrasion-resistant steel commonly used in the industry was selected. The same steel was used as a material for a washer onto which a padding weld El-Hard 63 (Elkrem Sp. z o.o., Toruń, Poland) containing an Fe–C–Cr alloy was applied. Abrasion-resistant (AR) steel Hardox 500 (SSAB, Oborniki, Poland) is characterised by high resistance to abrasive wear, high mechanical properties, and resistance to impact loads. It is used for the construction of machinery, including vehicles, and for operating parts processing an abrasive soil mass. In addition, this steel can be machined or welded. Due to its properties, this steel is used for operating parts of machines subjected to abrasive wear in a variety of mining and agricultural industries.

The chemical compositions of the test materials are provided in Table 1.

The steel in the as-delivered-by-the-manufacturer state, in the form of a 10 mm thick sheet, was subjected to surface hardfacing with the Fe–C–Cr alloy. Hardfacing was carried out using covered electrodes with a diameter of 3.2 mm. The hardfacing process was carried out in accordance with the manufacturer’s recommendations using a Dedra 180A inverter welding machine. A constant welding current (120 A) was maintained, with the electrode positively charged. The flat hardfacing position (PA) was applied. A single 4 to 5 mm thick padding weld layer was applied.

The hardness of the test materials was determined by the Vickers method in accordance with standard PN-EN ISO 6506 [25] using a Wilson VH1150 (Buehler, Waukegan Rd. Lake Bluff, IL, USA) hardness tester with a load of 98 N, which was maintained for 10 s. Microhardness measurements were taken for the padding weld. The microhardness testing was performed using an Innovatest 400-DAT (Innovatest, Horsham, PA, USA) hardness tester in accordance with standard PN EN ISO 6507-1 [26]. The testing was carried out at a load of 0.05 N.

To identify the particular phases, the chemical composition of the Fe–C–Cr alloy was analysed using the map method using a Phenom XL scanning electron microscope. The Phenom XL (PIK Instruments sp. z o.o., Piaseczno, Poland) microscope is equipped with an EDS analyser with an SDD detector (silicon drift detector), allowing for the detection of elements B to Cf. The EDS test results enabled the identification of carbide types, which were subsequently subjected to image analysis to determine their size, distribution, and geometric features.

Shape descriptors were used to describe the shape and size of the precipitates of the carbides found in the test materials. Only the descriptors that clearly differentiated the geometric features of the grains were selected. The characterisation of the carbide precipitates was performed based on the surface, roundness, corrugation coefficient, and orientation. The geometric features of the precipitates of individual carbides were determined by computer image analysis methods using Matlab Simulink 2020a software (MathWorks, Natick, MA, USA). The shape assessment was carried out using the functions presented in Table 2.

Surfaces were polished using an aqueous diamond slurry (DiaDuo Struers S.A.S., Champigny sur Marne, France) on a Struers LaboPol-5 polishing machine (Struers S.A.S., France), and samples for metallographic examination were additionally etched with nital (3% alcoholic HNO_3_ solution) following generally known procedures.

The microstructure images were taken using a Neophot 32 (Carl Zeiss Jena, Oberkochen, Germany) metallographic microscope. The photographs were taken at a magnification of 1000 times. Chemical analysis of the test materials provided the basis for identifying individual carbide precipitates. It was used as the basis for developing an algorithm to determine the geometric features of carbide-forming precipitates.

The condition of the test material surfaces following the tribological experiment was assessed using a Keyence VHX 7000 (Keyence, Mechelen, Belgium) microscope.

### 2.2. Laboratory Test

**Figure 1:**
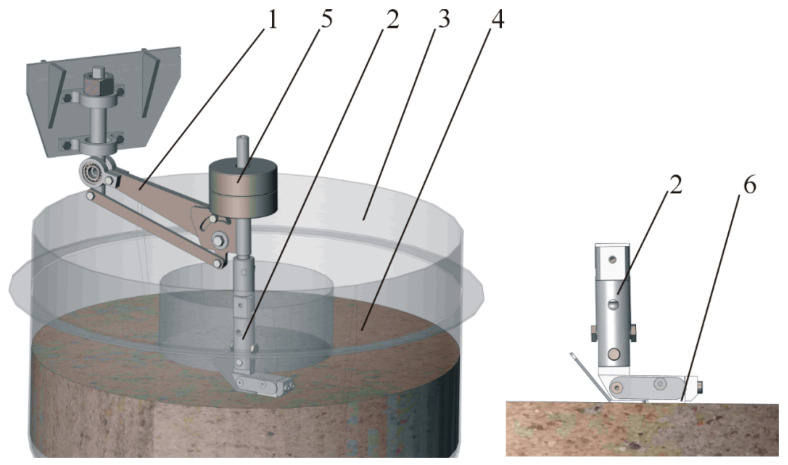
Test stand diagram: 1—rocker arm, 2—specimen holder, 3—bowl with abrasive mass, 4—abrasive mass, 5—loading mass, 6—specimen.

The testing under laboratory conditions was carried out at a “rotating bowl” test stand (Faculty of Technical Sciences, Olsztyn, Poland) used to assess wear in the soil mass (Figure 1).

During the experiment, a constant linear velocity of the samples of approx. 1.7 m·s^−1^ and a constant load of 50 N were maintained. The total friction distance for each specimen was 20,000 m. The specimens were weighed each time after a 2000 m friction distance had been covered. Mass wear measurements were taken every 2000 m using a laboratory balance with an accuracy of 0.0001 g.

Mass wear was calculated from the relationship:

*Z_w_* = *m_w_* − *m_i_* (1)

where:

*Z_w_*—mass wear [g];

*m_w_*—initial specimen weight before the wear testing [g];

*m_i_*—specimen weight after covering a specified friction distance [g].

To provide close-to-real conditions, the testing was performed in a natural abrasive mass, i.e., light clay. Grain size distribution was assessed by the laser diffraction method using a Mastersizer 2000 (Malvern Panalytical Ltd., Malvern, UK) laser particle composition meter based on PTG 2008, in accordance with standard ISO 14668-2(2017) [27] (Table 3).

Cuboid-shaped specimens with dimensions of 30 × 25 × 10 mm were cut out from the steel sheets. Test specimens were taken using the high-energy abrasive waterjet-cutting method to ensure no changes in the structure of the hardfaced steel. Then, the material was subjected to finishing using a flat-surface grinder to obtain a cuboidal form. A uniform roughness of the specimens was ensured by polishing at a level of Ra = 0.35–0.40 µm.

### 2.3. Operation Field Tests

Operation field tests were conducted to verify the suitability of using alloy additives to reinforce operating parts exposed to intensive abrasive wear. For the operation field tests, rigid cultivator tines, both in the as-delivered state and hardfaced with an Fe–C–Cr-based electrode, were used. The tests were carried out at a working depth of approx. 0.15 m and a speed of 1.5 m·s^−1^.

Under field conditions, the tests were conducted in the same soils as those used in the laboratory tests.

Before and after the tests, changes in the geometry of the operating parts were identified using the 3D scanning technique. This technique enabled the determination of changes to the tine shape and the quantification of the intensity of wear of selected areas of the test operating parts. The 3D scanning was carried out using an Atos Core optical 3D scanner manufactured by GOM^®^ with an accuracy of 0.02 mm. The results obtained were processed using the GOM Inspect^®^ software (2018 hot fix 4, rev. 114961).

The scanning process used triangulation, i.e., obtaining point clouds from the surface. During the scanning process, it was necessary to place markers on the object, which helped to merge individual scans taken at different angles. On this basis, a three-dimensional model was generated [28]. In a further step, a network of triangles was prepared to obtain an STL model [29].

**Figure 2:**
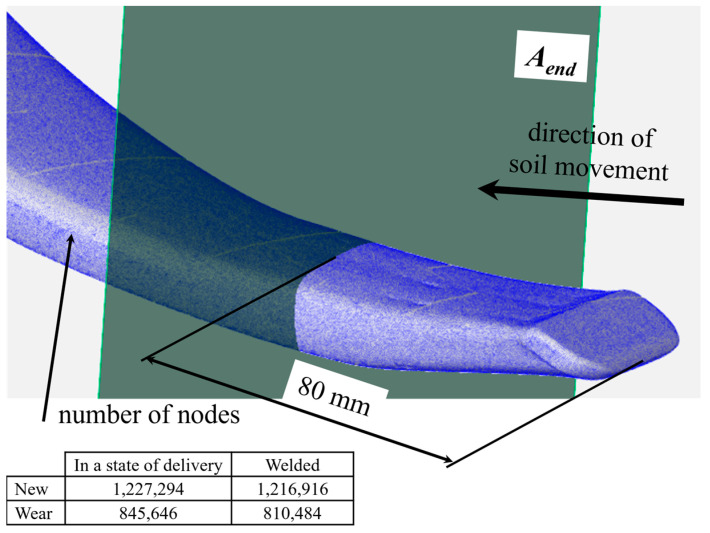
Tine surface and position of the A_i_ plane.

Figure 2 provides the number of network nodes that represent the actual surface used for analysing the wear of the operating part. Wear analysis was carried out on the front part of the tine, which is most exposed to the impact of the abrasive environment. In this part of the tine, the greatest force impact on the soil occurs. The total loss of volume after ploughing an area of approx. 24 ha was used as a measure of wear.

The tine area subjected to wear analysis was limited by the front edge of the tine on one side and the surface A_end_ located 80 mm from the front edge of the tine on the other side (Figure 2). The surface A_end_ was oriented perpendicular to the plane located on the flat surface of the lower part of the tine. In the next step, this area was divided into elementary volumes that were limited by planes A_i_ (Figure 2) running parallel to plane A_end_, which were located at 1 mm intervals over a distance of 80 mm. The position of the operating part’s surface points on planes A_i_ enabled the determination and comparison of the surface profile of the scanned parts.

## 3. Results

### 3.1. Laboratory Test Results

**Figure 3:**
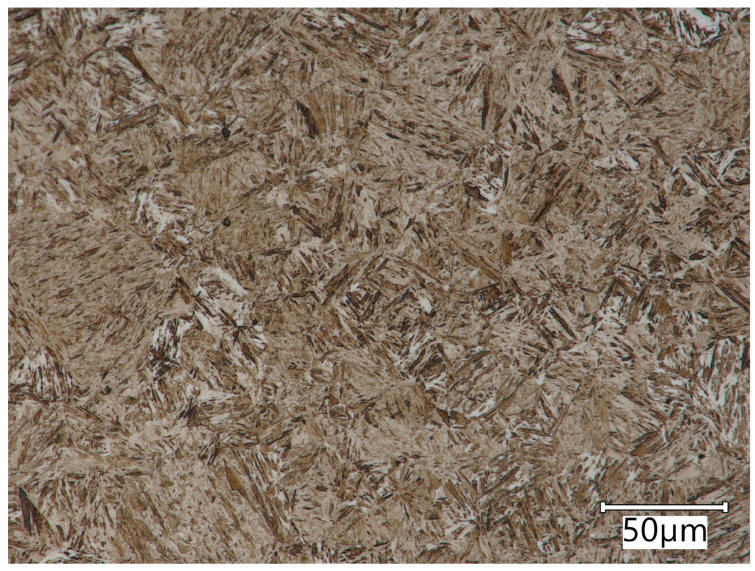
AR steel microstructure. Microstructure of tempered martensite with former austenite grain boundaries. Light microscopy. Etched with 3% HNO_3_.

The AR steel tested (Figure 3) is characterised by a microstructure of tempered martensite (brown areas) with bainite (light acicular areas). The microstructure can be described as similar to that of tempering sorbite. Furthermore, it should be noted that this steel is characterised by a structure with a post-martensitic orientation, with precipitates of small carbides distributed coherently within the martensite grains.

**Figure 4:**
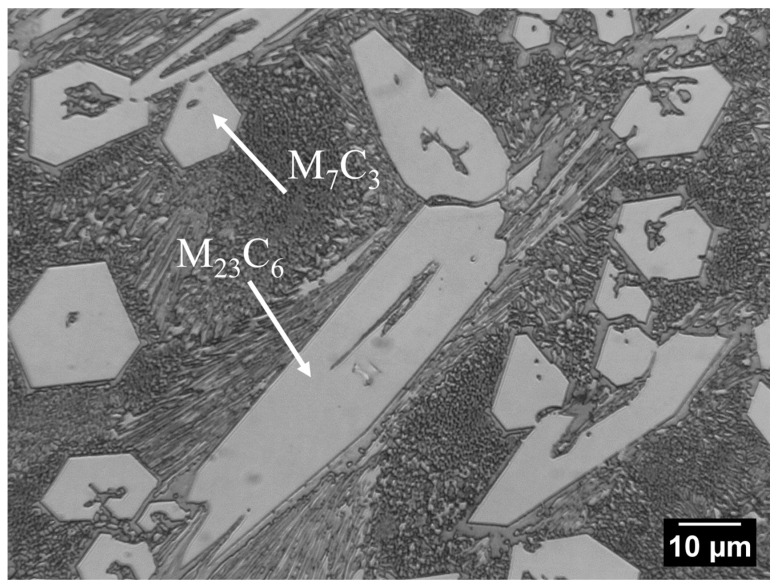
Fe–C–Cr alloy microstructure: Alloy ferrite with precipitates of chromium carbide and large precipitates of primary M_7_C_3_ carbides (Fe,Cr_7_C_3_) against the background of mixed alloy ferrite and M_23_C_6_ carbides; light microscopy.

The Fe–C–Cr alloy microstructure is characterised by large precipitates of primary chromium carbides in a matrix of mixed alloy ferrite and carbides (Figure 4). Fe–C–Cr alloys containing M_7_C_3_ carbide are widely used as materials for which heat and wear resistance is required [30]. Based on the relevant literature, it can be concluded that the M_7_C_3_ carbide has three structures: triangular, rhombic, and hexagonal [31]. Abrasive wear resistance is closely linked to the morphology and presence of M_7_C_3_ carbide in these materials. These carbides contribute to limiting the direct contact between the material and the abrasive particles, thus reducing wear. The resistance to abrasive and erosive wear is determined by the volumetric proportion of hard carbides. Due to the properties of the carbides contained in Fe–C–Cr alloys, they are used for operating parts exposed to intensive abrasive wear. These alloys are also used as hardfacing materials. As for hardfacing with high-chromium materials, these alloys are in a super-eutectic state, in which M_23_C_6_ carbides are surrounded by a eutectic structure, which contributes to a reduction in cracks. As noted by the authors [32], two abrasive wear-resistant components present in the microstructure play different roles: hard carbide precipitates limit the formation of grooves by abrasive particles, and a durable matrix ensures appropriate bonding and resistance of the microstructure to cracking.

If high-chromium alloys (Fe–C–Cr) for hardfacing have a super-eutectic structure (the primary M_23_C_6_ is surrounded by Cr–Fe and M_23_C_6_, a eutectic structure), they contribute to a reduction in the occurrence of cracks. This is determined by a lamellar eutectic structure that is resistant to the propagation of cracks along the grain boundary. Hardening alloys obtained using high-energy-density sources, such as electron beam welding, plasma arc, and laser, are widely used to increase the resistance of material surfaces to wear and corrosion.

The results of washer and padding weld hardness measurements are provided in Table 4.

The microhardness measurement results are provided in Table 5.

**Figure 5:**
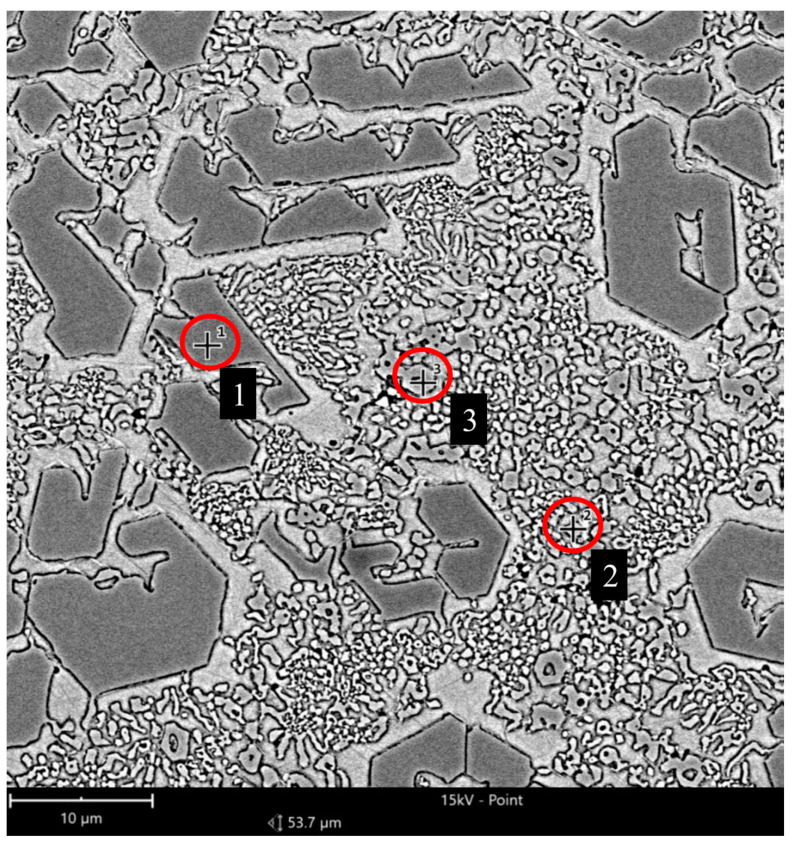
Fe–C–Cr alloy microstructure subjected to chemical analysis; 1, 2, 3—measurement points for chemical composition.

**Figure 6:**
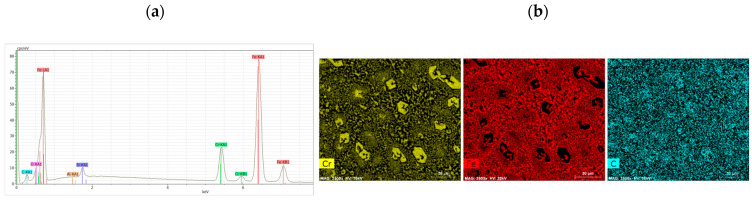
EDS analysis results from the areas shown in the Fe–C–Cr alloy microstructure image. (**a**) X-ray spectrum, (**b**) map of the distribution of elements.

The results of analysis of the Fe–C–Cr alloy chemical composition, performed by the EDS method, are provided in Figure 5 and Table 6. The microstructure exhibits precipitates of primary M_7_C_3_ chromium carbides deposited in a matrix of mixed alloy ferrite and M_23_C_6_ carbides. The map of the distribution of individual elements in the padding weld is provided in Figure 6.

Figure 6 shows the EDS analysis results from the areas shown in the Fe–C–Cr alloy microstructure image:

Image analysis was carried out to verify the chromium carbide grain distribution and size. For the analysis, shape descriptors that describe the geometric features of chromium carbide precipitate grains were selected. The results are provided in Table 7.

**Figure 7:**
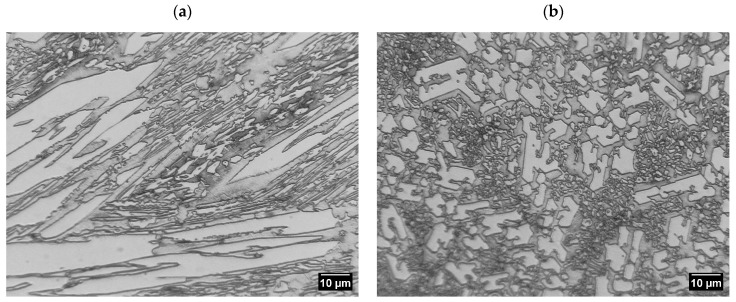
Precipitation of chromium carbides in the Fe–C–Cr-based weld deposit; (**a**) in the longitudinal orientation (longitudinal), (**b**) in the transverse orientation; 1000× magnification.

The Fe–C–Cr-based padding weld was characterised by a non-uniform shape of chromium carbide precipitates depending on the area observed on the specimen surface (Figure 7a). The shape of the resulting carbide phases might have been influenced by eutectic transformations taking place during crystallisation. As noted in the literature [33], the shape and morphology of chromium carbides in Fe–C–Cr alloys are influenced by the crystallisation temperature and the alloy cooling rate. The literature (as described above) indicates that when hardened at a temperature of 1100 °C, M_7_C_3_ eutectic carbides with a strip shape and a discontinuous mesh distribution precipitate from the microstructure of retained austenite and martensite. At a temperature of 900 °C, network-shaped secondary carbides also precipitate. Similar observations were made in [34] when examining layers welded onto high-chromium white iron. It was noticed that in single-layer welded coatings, twice the amount of heat input per unit length caused a change in the solidification mode from hypoeutectic to almost eutectic. This resulted in an increase in the volume fraction of Cr carbides and an increase in the roundness parameter of the precipitates. In the double layer, the same heat input does not significantly affect the volume fraction of carbides. However, the values of all morphological parameters undergo noticeable changes. The sizes of primary and eutectic carbides are approximately four and two times larger, respectively. Primary Cr carbides have a blade-like appearance, and eutectic Cr carbides have a needle-like appearance. The roundness parameter is reduced three times. By increasing the carbon and chromium contents, the blade-like morphology of the original Cr carbides is replaced with a rod-like morphology. This process results in further crystallisation into primary carbides and the formation of separate acicular precipitates (Figure 7b).

This may be due to the fact that large precipitates of primary carbides can already be formed in the liquid, while smaller ones are formed as a result of eutectic transformation. The smaller carbides partially further crystallise into primary carbides and partially form separate precipitates, which take on an acicular shape [32].

**Figure 8:**
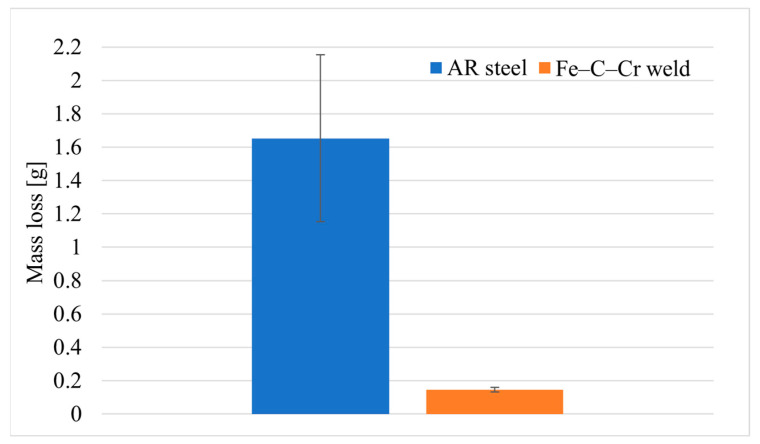
Loss of weight of test materials.

The results of the test material weight loss after covering a friction distance of 20,000 m are provided in Figure 8.

Based on the results obtained from laboratory tests, it was noted that AR steel exhibited 14 times greater wear than the Fe–C–Cr-based padding weld. This wear is undoubtedly affected by alloy additives, which, for the padding weld, are chromium additives.

**Figure 9:**
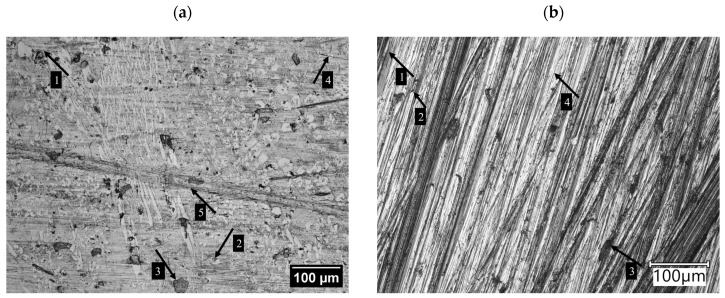
Surface of the tested materials after wear: (**a**) overlay based on Fe–C–Cr; (**b**) AR steel; 1—ploughing, 2—micro-cutting, 3—spalling, 4—scratching, 5—ridging.

The microstructure image shows that soil mass had a destructive effect mainly on the matrix of the material (Figure 9a), whereas in the areas with high concentrations of chromium precipitates, this effect was significantly weaker (Figure 9b).

The main wear mechanisms on the AR steel surface include scratching (4) and ploughing (1). Local micro-cutting (2) and spalling (3) of the material are also caused by micro-fatigue resulting from the cyclic impact of contact stresses on the surface layer of the material due to the impact of sand grains.

For the Fe–C–Cr-based padding weld, ploughing (1), scratching, (4) and micro-cutting (2) of the matrix were observed. Also, spalling (3) due to the impact of quartz grains was identified, which is a characteristic phenomenon in the wear process in soil mass with a high sand fraction. Moreover, the spalling of chromium carbides (3) and craters after the release of the full precipitates of individual phases were identified. In the direction opposite to the chromium carbide alignment, ridging was observed (5).

The grooves were most intense in the matrix area (1), whereas the firmly embedded carbides remained intact (2). The poorly embedded carbides were entirely removed (3) by being pulled out from their matrix under the influence of abrasive grains, which undoubtedly contributed to the weight loss of this material. The areas left by the removed carbides were similar in shape to the carbides.

**Figure 10:**
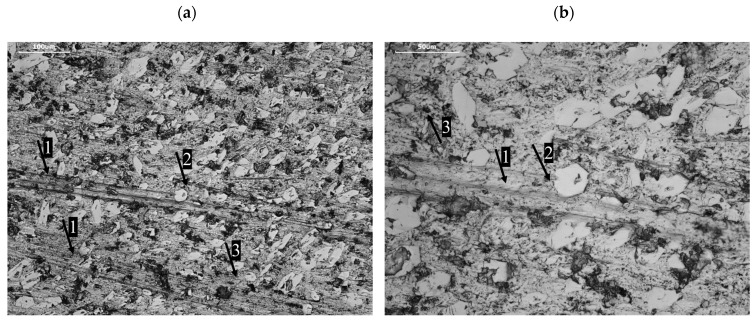
Surface of the tested materials after wear: (**a**) Fe–C–Cr-based weld; (**b**) enlargement of the area marked in (**a**). 1—grooves, 2—carbides participate, 3—spall after removed carbide.

This course of the wear process could have been significantly affected by the carbide precipitates of individual phases. The area occupied by the chromium carbide precipitates was larger than 341 μm^2^. The chromium carbide grains were characterised by an octagonal shape (Figure 10b), as evidenced by the low roundness coefficient values (0.56). Moreover, the acicular precipitates were arranged in line with the direction of the abrasive mass impact, which results from the orientation coefficient value (3°). The increase in the resistance of the test hardfaced layer to the impact of abrasive grains is undoubtedly contributed to by the corrugation coefficient of the carbide phases. The compact chromium carbide precipitates with no sharp edges cause the abrasive grains to roll along their circumference and only remove the soft matrix.

### 3.2. Operational Testing Results

The hardness measurement results for the cultivator tines used in the testing are provided in Table 8.

Original operating parts made from abrasion-resistant steel were used for the testing. The reduced hardness of the hardfaced layer on the tines is related to the washer material. The washer used in laboratory testing was characterised by a hardness of approx. 500 HV10, whereas the hardness of cultivator tines was approx. 400 HV10. The hardness of the hardfaced layer is also determined by the hardness of the washer material.

**Figure 11:**
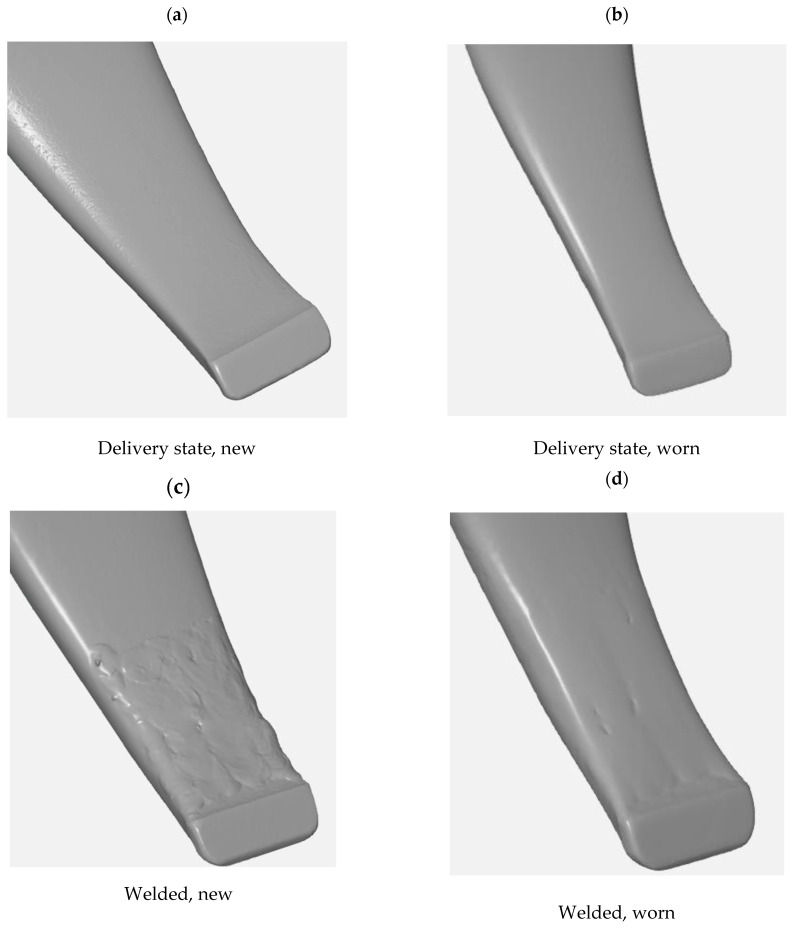
The cultivator tines used for testing: (**a**) as-delivered before the testing, (**b**) as-delivered following the testing, (**c**) hardfaced before the testing, (**d**) hardfaced following the testing.

Figure 11 shows a view of 3D scans of the front part of the cultivator tines before and after operation field testing.

**Figure 12:**
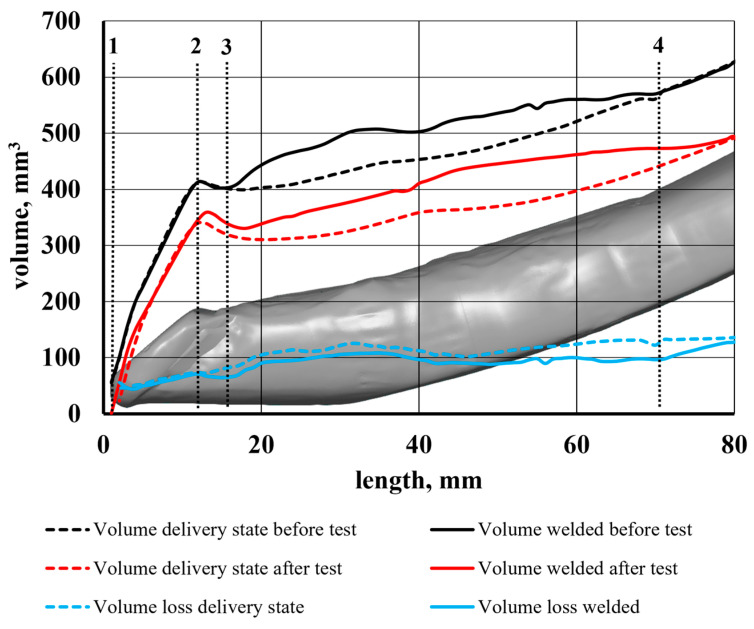
Volumetric wear characteristics. 1, 2–the surface of the sintered carbide blade, 3, 4–the hardfaced part of the tine.

Figure 12 shows the volumetric wear characteristics determined from the 3D image analysis. Section 1 and Section 2, as indicated in the figure, refer to the surface of the sintered carbide blade. The tested sections for material and geometric characteristics were the same for the tines being compared. Section 2 and Section 3 are the top surface of the carbide blade, whereas Section 3 and Section 4 cover the hardfaced part of the tine.

A comparison of the volumetric wear characteristics (Figure 12) showed that the volumetric wear of the front part of the tine reinforced with a sintered carbide plate was almost identical in the test tines. The hardfacing process carried out before the operation field tests on Section 1 and Section 2 resulted in an increase in the tine volume by approx. 2522 mm^3^. The addition of this volume helped minimise the wear of the base material of the tine in this section. The wear on other sections of the tested front part of the tine also exhibited similar values.

**Figure 13:**
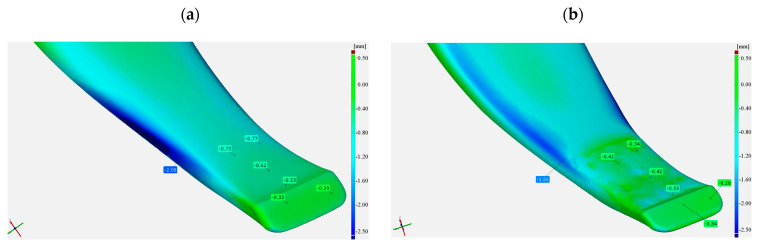
A view of the tine surface following testing; (**a**) in the as-delivered state, (**b**) hardfaced.

As follows from the material loss analysis, the wear of the analysed tines is unevenly distributed across their cross-sections. In both cases, significant material losses can be seen on the side (non-hardfaced) surfaces (Figure 13).

**Figure 14:**
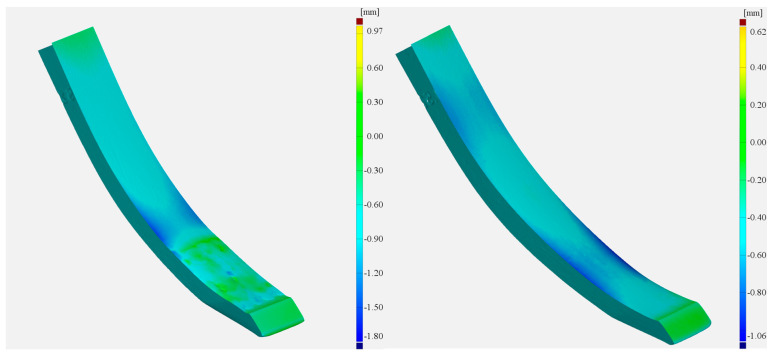
Test tine parts subjected to analysis.

As this study is concerned with the properties of the hardfaced layers, it was the central parts of the tines, with a width of 30 mm, located along the tine’s axis of symmetry, that were subjected to further analysis (Figure 14).

**Figure 15:**
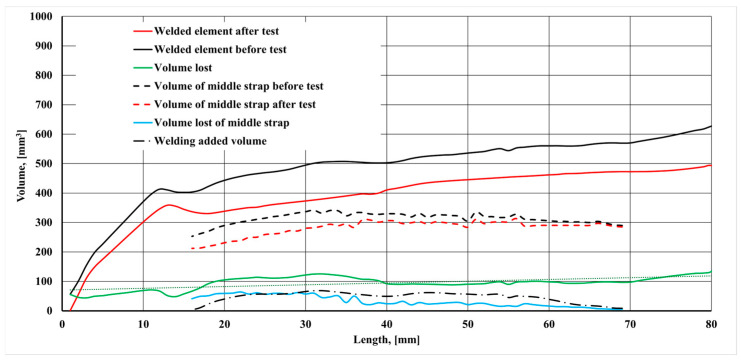
Volumetric characteristics of the central section of the hardfaced tine.

Figure 15 shows a loss of volume of the hardfaced tine.

**Figure 16:**
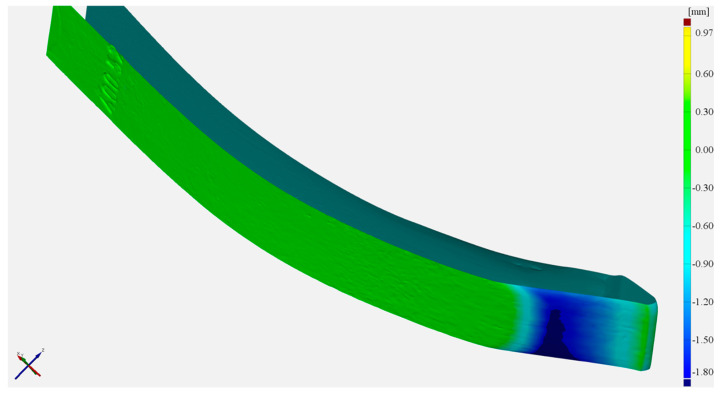
Material losses on the lower surface of the tine.

**Figure 17:**
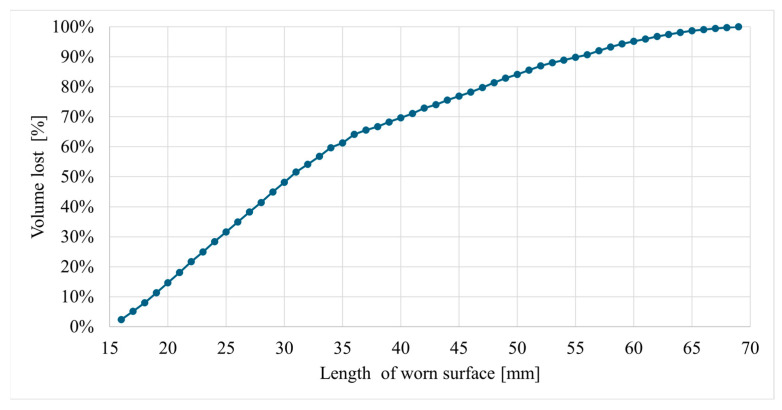
Cumulative loss of the padding weld volume.

The greatest wear was noted on a section from approx. 16 mm to approx. 40 mm from the front edge of the tine. This is because in this section, there was wear on the lower surface of the tine. This process results from the tine self-sharpening. The volume loss seen in the figure is the sum of the loss of the padding weld and of the lower part of the tine (Figure 16). Most manufacturers are limited to surfacing the upper working surface of the tooth and, as Figure 17 shows, significant wear also occurs on the surface opposite it.

The cumulative loss of the padding weld volume over the entire length is shown in Figure 17.

It follows from the analysis of the cumulative loss of the padding weld volume that in the section of 16–40 mm, there is a loss of approx. 70% of the total padding weld volume in the central part of the tine. In this part of the tine, the abrasive wear process is more intense than in the 40–70 mm section. It is worth noting that the working position of the tine results in the smallest interaction angles of abrasive particles on the surfaces of the tine sections with the highest wear. As the distance from the tine apex increases, the angle between the direction of movement and the tine surface also increases. An increase in the particle contact angle is accompanied by a decrease in wear intensity. As noted by Zum Gahr [35], an increase in the contact angle is associated with a decrease in the proportion of ploughing and micro-cutting in favour of erosive wear. Pure micro-cutting results in a volume loss by chips equal to the volume of the wear grooves. Microcracking as an effect of erosive wear occurs when highly concentrated stresses are imposed by abrasive particles, particularly on the surface of brittle materials [35]. The erosive wear of steel results in less material loss compared to ploughing and micro-cutting. However, as Choteborsky writes, volume loss can occur due to the action of multiple abrasive particles or the repeated action of a single particle. The material may be repeatedly torn aside by passing particles and may break away as a result of low-cycle fatigue, i.e., micro-fatigue [36]. Choteborski’s observations [36] indicating the dominance of furrowing and microcracking processes in the case of ductile materials and of microcracking and spalling in the case of brittle materials are confirmed by the findings presented in this study. Furrowing and microcracking processes dominate in the base and matrix material, while fatigue chipping dominates in the surface layer.

**Figure 18:**
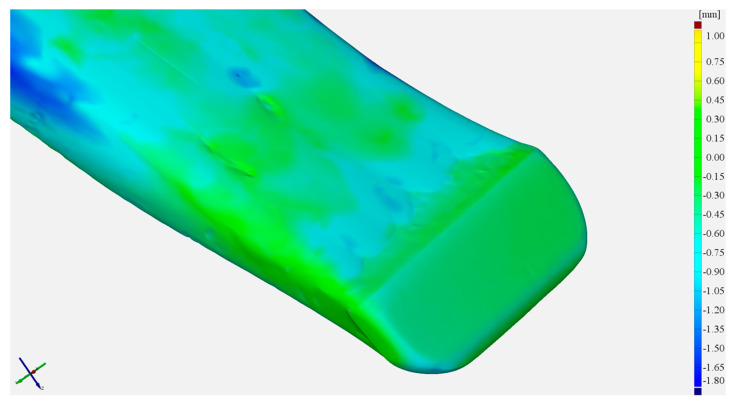
View of the hardfaced tine surface.

When analysing the loss of the hardfaced layer volume (Figure 15), one can note an area along the length of the operating part (16–21 mm) where the volume loss is greater than the volume added in the hardfacing process. This is due to the fact that a part of the padding weld, the volume of which includes the total volume of the padding weld, was cut off along with the cut-off parts of the tine. In addition, it can be noted that the front edge of the padding weld is unevenly distanced from the tine blade edge (Figure 18). This fact contributes to a lower value of the volume added in this area. Moreover, the non-hardfaced surfaces were subject to more intensive wear, which inflated the total loss of volume of this area.

**Figure 19:**
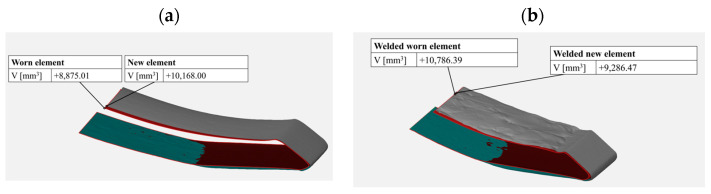
Comparison of the volume of the tested elements; (**a**) in the delivered state, (**b**) the welded tine.

The test results showed that within the area of the tine subjected to hardfacing (Figure 19b), the material loss was lower than that for the same area of the tine in the as-delivered state (Figure 19a), which is indicative of the positive effect of hardfacing on abrasive wear resistance. By comparing the models obtained from 3D scanning, it is possible to identify the critical areas of the working surfaces that require reinforcement by hardfacing. The beneficial effect of using Fe–C–Cr alloy-based reinforcement and regeneration layers is confirmed by the lower volumetric wear of the hardfaced operating part as compared to that of the tine in the as-delivered state. For the hardfaced tine, a 7% loss of volume was noted in relation to the operating part before the testing and following the friction process. However, for the operating part in the as-delivered state, this difference amounted to 12%.

**Figure 20:**
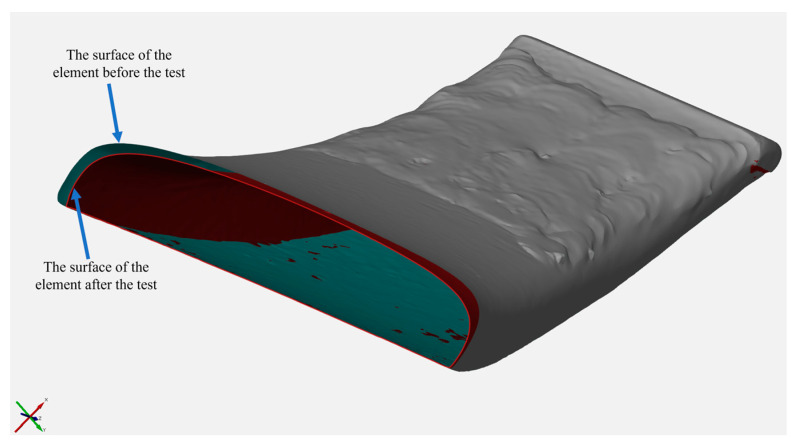
View of the hardfaced tine before and after the wear testing.

Having considered the volume loss, the results are not that unambiguous. A loss of the tine volume, in addition to the top hardfaced layer, also occurs on the side and the lower surfaces. The wear of non-reinforced areas significantly contributes to a loss of volume of the entire operating part. The wear is particularly concentrated on the side edges of the tine (Figure 20).

The results obtained show that the hardfaced layers contribute to an increase in the durability of the parts operating in the abrasive soil mass. However, in order to achieve the maximum effect, not only the top surface but also the side surfaces and the lower surface in the front of the operating part must be subjected to hardfacing. This action will prevent a self-sharpening effect, i.e., the loss of material on the surface located opposite the main cutting surface.

## 4. Conclusions

1. The paper takes an original approach to increasing the durability of soil working elements. In the first stage, based on 3D scanning, which has been used in previous studies to develop numerical models of force distribution during machining, the surfaces with the highest wear intensity under light clay conditions were determined. A Fe–C–Cr-based surfacing was applied to these surfaces, which is characterised by the simplicity of application and good technological properties. This approach made it possible to reduce the cost of the technology by reducing the surfacing and increasing the wear resistance of the surfaced coulters compared to the original ones.
2. It follows from the analysis of the cumulative loss of the padding weld volume that in the section located 16–40 mm of the front edge of the tine, there is a loss of approx. 70% of the total padding weld volume in the central part of the tine. In this part of the tine, the abrasive wear process is more intense than in the section at 40–70 mm. When analysing the loss of the hardfaced layer volume, one can note an area along the length of the operating part (16–21 mm) where the volume loss is greater than the volume added in the hardfacing process.
3. An Fe–C–Cr alloy padding weld was characterised by a significantly lower loss of weight and volume as compared to the washer material and the tine in the as-delivered state. As for the laboratory tests, the loss of the washer material weight was 14 times greater than that for the hardfaced layer. In operation field testing, the loss of non-hardfaced tine volume was approx. 1.3 times higher than that for hardfaced tine. It is therefore apparent that carbide phases prevent the destruction processes, which contributes to a decrease in the wear intensity, as compared to that for steel.
  The AR steel tested was characterised by a microstructure containing tempered martensite with bainite precipitates. The Fe–C–Cr-based alloy was characterised by a sub-eutectic, eutectic, and super-eutectic structure with a heterogenous shape of chromium carbide precipitates with a sub-eutectic structure. The acicular chromium carbide grains were positioned in line with the direction of the abrasive mass impact, as evidenced by the value of the orientation coefficient (3°), which decisively contributed to an increase in the resistance of the tested hardfaced layer in relation to the impact of the abrasive grains.
4. In laboratory tests, only the welded layer was subjected to abrasive wear, while the unhardened sides of the sample were shielded. However, in field tests, all tooth surfaces, including unhardened surfaces, were subjected to abrasive wear.
  Therefore, to increase the durability of consumable parts, it is important to define and surface the surfaces exposed to the greatest wear. Thus, the information presented in the literature that tests under laboratory conditions should be used as preliminary results was confirmed. The final information on the anti-wear properties of materials used for working elements in the soil is obtained during tests in actual operation, in this case under light clay conditions.
5. Wear processes characteristic of abrasive wear are dominant in the test materials.
  On the surface of the test materials, typical abrasive wear mechanisms are evident, primarily ridging and micro-cutting. The fatigue of the material surface, as well as spalling due to the impact of quartz grains, were also identified. In addition, on the padding weld surface, chromium carbides were spalling, and all precipitates of individual phases were coming out.
6. A comparison of the volumetric characteristics of the test operating parts showed that the volumetric wear of the front part of the tine reinforced with a sintered carbide plate was almost identical. The addition of volume through hardfacing helped minimise the wear of the base material of the tine. As follows from the material loss analysis, the wear of the analysed tines is unevenly distributed across their cross-section. In both cases, significant material losses can be seen on the side surfaces that were not hardfaced.
7. The results show that the method used to determine the wear characteristics in the volumes bounded by the incisal planes of the teeth makes it possible to take into account the technological factor (the angle of the teeth in the cultivator) affecting wear. Further research should be carried out to identify zones that require reinforcement to reduce damage to working parts while taking into account the properties of the soil abrasive compound. It should be noted that depending on the shape of the working part and its purpose, the areas subject to the most wear vary.

## Figures and Tables

**Table 1:** Chemical compositions of the test materials.

Material	% Elemental Content								
	C	Cr	Mn	Si	Mo	Ni	P	S	B
AR steel (Hardox 500)	0.29	1.00	1.60	0.70	0.60	0.50	0.02	0.01	0.004
Padding weld Fe–C–Cr (El-Hard 63)	4.50	34.00	-	1.00	-	-	-	-	-

**Table 2:** Shape descriptors used for carbide precipitates.

Shape Descriptor	Description of the Parameter (Descriptor)
(Surface) area	Area of the projection of the particle on the surface
Roundness	Calculated from the following relationship: $round=\frac{4\pi \times F}{U^{2}}$
Corrugation coefficient	$W=\frac{U_{w}}{U_{g}}$ *U_w_*—length of the convex object’s perimeter; *U_g_*—length of the object’s boundary perimeter
Orientation	The angle between the x-axis and the larger axis of the ellipse; it takes on values ranging from −90° to 90°

**Table 3:** Soil mass characteristics.

% Fraction Content mm		
Silt <0.002	Dust 0.002–0.050	Sand 0.050–2.000
7.02	40.23	52.66

**Table 4:** Test material hardness measurement results.

Material	Hardness [HV10]	Std. Dev. [HV10]
Hardox 500	500–562	20.7
El-Hard 63	830–1232	120.3

**Table 5:** Fe–C–Cr padding weld microhardness measurement results.

Material Fe–C–Cr	Average Microhardness [HV0.05]	Minimum [HV0.05]	Maximum [HV0.05]
Matrix	872	863	891
Chromium carbide	1515	1314	1702

**Table 6:** Atomic concentration of the elemental content in the chemical composition of the weld tested.

	C	Cr	Fe	Si
	[% Atomic Concentration]			
Point 1	81.66	11.35	6.99	0.54
Point 2	80.73	9.00	10.26	0.49
Point 3	80.63	10.18	9.18	0.52

**Table 7:** Summary of chromium carbide shape descriptor values.

Shape Descriptor	Value
Area [µm^2^]	341.76
Circularity [-]	0.56
Corrugation coefficient, convexity [-]	0.66
Orientation [°]	3.00

**Table 8:** Hardness measurement results for the test cultivator tines.

Specimen	Hardness [HV10]	Std. Dev. [HV10]
Welded	743	16
Nominal	392	8

## Data Availability

The original contributions presented in the study are included in the article, further inquiries can be directed to the corresponding author.
